# The Metabolic Response to a Low Amino Acid Diet is Independent of Diet-Induced Shifts in the Composition of the Gut Microbiome

**DOI:** 10.1038/s41598-018-37177-3

**Published:** 2019-01-11

**Authors:** Heidi H. Pak, Nicole E. Cummings, Cara L. Green, Jacqueline A. Brinkman, Deyang Yu, Jay L. Tomasiewicz, Shany E. Yang, Colin Boyle, Elizabeth N. Konon, Irene M. Ong, Dudley W. Lamming

**Affiliations:** 1 William S. Middleton Memorial Veterans Hospital, Madison, WI USA; 2 Department of Medicine, University of Wisconsin-Madison, Madison, WI USA; 3 Interdisciplinary Graduate Program in Nutritional Sciences, University of Wisconsin-Madison, Madison, WI USA; 4 Endocrinology and Reproductive Physiology Graduate Training Program, University of Wisconsin-Madison, Madison, WI USA; 5 Molecular and Environmental Toxicology Program, University of Wisconsin-Madison, Madison, WI USA; 6 Department of Obstetrics and Gynecology, University of Wisconsin-Madison, Madison, WI USA; 7 Department of Biostatistics and Medical Informatics, University of Wisconsin-Madison, Madison, WI USA; 8 University of Wisconsin Carbone Comprehensive Cancer Center, University of Wisconsin, Madison, WI USA

## Abstract

Obesity and type 2 diabetes are increasing in prevalence around the world, and there is a clear need for new and effective strategies to promote metabolic health. A low protein (LP) diet improves metabolic health in both rodents and humans, but the mechanisms that underlie this effect remain unknown. The gut microbiome has recently emerged as a potent regulator of host metabolism and the response to diet. Here, we demonstrate that a LP diet significantly alters the taxonomic composition of the gut microbiome at the phylum level, altering the relative abundance of Actinobacteria, Bacteroidetes, and Firmicutes. Transcriptional profiling suggested that any impact of the microbiome on liver metabolism was likely independent of the microbiome-farnesoid X receptor (FXR) axis. We therefore tested the ability of a LP diet to improve metabolic health following antibiotic ablation of the gut microbiota. We found that a LP diet promotes leanness, increases energy expenditure, and improves glycemic control equally well in mice treated with antibiotics as in untreated control animals. Our results demonstrate that the beneficial effects of a LP diet on glucose homeostasis, energy balance, and body composition are unlikely to be mediated by diet-induced changes in the taxonomic composition of the gut microbiome.

## Introduction

Around the world, approximately 425 million people have diabetes, and that number is expected to grow by 50% over the next three decades^1^. Beyond the direct effects of diabetes on mortality, its impact is amplified by its association with other causes of morbidity and mortality, such as cardiovascular disease^2^, cancer^3^, and Alzheimer’s disease^4^. Type 2 diabetes, which is associated with diet and obesity, accounts for the vast majority of diabetes cases, and the epidemic rise in obesity has fueled the development of this health crisis.

Dietary interventions to control or prevent type 2 diabetes could be highly effective and affordable, but long-term reduced calorie diets have not proven to be sustainable for most people. Diets that alter the level of specific macronutrients without a decrease in caloric consumption may be more sustainable^5^; one variety of such diets are high protein, low carbohydrate diets such as the Atkins diet, which promise rapid weight loss without restricting calories^6^. Some clinical trials have observed that high protein diets can promote weight loss^7–9^, at least in highly compliant subjects^10^. However, long-term prospective cohort studies have observed that high protein consumption is associated with increased insulin resistance, diabetes, cancer, and cardiovascular disease, and an overall increase in mortality^11–13^.

In agreement with these findings, recent long-term studies in *Drosophila* and mice, as well as a short-term randomized control trial conducted in humans, find that low protein (LP) diets are associated with improvements in health, survival, and insulin sensitivity^13–18^. Reducing dietary protein largely blocks the effect of a high-fat diet on glucose tolerance^18^, and we recently showed that in a mouse model of pre-existing diet-induced obesity, reducing dietary protein rapidly restored metabolic health, dramatically reduced adiposity, and improved glucose tolerance and insulin sensitivity^19^. While some of these phenotypes are mediated in part by the insulin-sensitizing and energy expenditure promoting hormone fibroblast growth factor 21 (FGF21), it is likely that other mechanisms are also involved^18–24^.

Over the last decade, numerous studies have found that the composition of the gut microbiome plays an important role in regulating the metabolic health of both rodents and humans^25,26^ by mediating the response to drugs, diet, and aging^27–33^. One major pathway by which the gut microbiota regulates glycemic control is by altering bile acid metabolism and activating the farnesoid X receptor (FXR) – FGF15 signaling axis^34,35^. Recent work suggests that at least in rodents, the major dietary factors that regulate the taxonomic composition of the gut microbiome are protein and carbohydrate intake^36^. However, the source of dietary protein – e.g. red meat, white meat, dairy, or plant protein – also has an important effect on the taxonomic composition of the gut microbiome^37^. It remains unknown if the effect of a LP diet on the composition and function of the gut microbiome plays a role in its beneficial metabolic effects.

In this study, we determined that an amino acid defined LP diet, which has similar metabolic benefits to a LP diet containing natural protein^22^, alters the taxonomic and functional composition of the gut microbiome. We found that a LP diet significantly alters the hepatic transcriptome, possibly reducing FXR-FGF15 signaling. Finally, we found that ablation of the gut microbiome with antibiotics does not significantly alter the metabolic response to a LP diet. Our data suggests that while dietary protein plays an important role in shaping the taxonomic and functional composition of the gut microbiome, these diet-induced changes do not mediate the beneficial metabolic effects of a LP diet in young, lean mice.

## Materials and Methods

### Animals and Treatments

For all experiments, male C57BL/6J mice were purchased from The Jackson Laboratory and group housed in static microisolator cages in a specific-pathogen free animal facility. For experiments investigating the composition of the gut microbiome and transcriptional profiling of the liver, mice were purchased at 8 weeks of age, and diet changes occurred at 9 weeks of age. Approximately 4 months later, cecal contents and livers were collected from mice sacrificed in the morning following an overnight, approximately ~16 hr fast. For experiments in which the gut microbiome was ablated with antibiotics, mice were purchased at 5 weeks of age; starting at 6 weeks of age, mice were randomized at the cage level to receive water or water containing antibiotics as described below. Diet changes occurred at 9 weeks of age, and antibiotic treatment was continued for the duration of the experiment. All procedures involving animals were approved by the Institutional Animal Care and Use Committee of the William S. Middleton Memorial Veterans Hospital (Madison, WI), and all experiments were performed in accordance with relevant guidelines and regulations.

### Diets

Prior to 9 weeks of age, animals were fed the standard facility chow (Purina 5001; Purina Mills, Richmond, IN, USA). Amino acid defined animal diets (non-irradiated) were obtained from Envigo (formerly Harlan Laboratories). At 9 weeks of age, animals were switched to either a Control amino acid defined diet (TD.140711; 22.0% of calories derived from amino acids; 59.4% from carbohydrate; 18.6% from fat) or a Low Protein amino acid defined diet (TD.140712; 7.1% of calories derived from amino acids; 74.4% from carbohydrates; 18.5% from fat). The complete composition of these diets has been previously described^22^.

### Antibiotic Treatment

The gut microbiome was ablated using an antibiotic treatment protocol previously described to efficiently ablate the gut microbiome of mice^38^. Briefly, mice were provided with free access to autoclaved water containing 1 g/L ampicillin, 500 mg/L vancomycin, and 1 g/L neomycin; however, in contrast to the protocol followed in^38^, aspartame was omitted due to its negative effects on glucose homeostasis and body composition in mice^39^. The mice and the water bottles were weighed and changed biweekly to monitor water intake. Control mice were provided with autoclaved water not containing antibiotics. To verify the efficacy of the antibiotic treatment, fecal pellets were collected and total DNA was extracted using a modification of a previously described protocol^40^. Briefly, fecal pellets (~30–50 mg) were resuspended in a solution containing 500 µL of extraction buffer [200 mM Tris (pH 8.0), 200 mM NaCl, 20 mM EDTA], 210 µL of 20% SDS, 500 µL phenol:chloroform:isoamyl alcohol (pH 7.9, 25:24:1) and 500 µL of Fisher Scientific 1.4 mm diameter ceramic beads (Cat# 15340159). Following mechanical disruption using a FastPrep 24 (M.P. Biomedicals), the solution was centrifuged at 8,000 rpm at 4 °C for three minutes. The aqueous layer was then sequentially precipitated using sodium acetate/isopropanol and sodium acetate/ethanol. DNA samples were then quantified using a Nanodrop 2000c.

### Mouse metabolic phenotyping

Glucose and alanine tolerance tests (GTT and ATT) were performed by fasting the mice overnight for 16 hr and then injecting either glucose (1 g/kg) or alanine (2 g/kg) intraperitoneally as previously described^41,42^. Glucose measurements were taken using a Bayer Contour blood glucose meter and test strips. Mouse body composition was determined using an EchoMRI 3-in-1 Body Composition Analyzer. For assay of multiple metabolic parameters (O_2_, CO_2_, food consumption) and activity tracking, mice were acclimated to housing in a Columbus Instruments Oxymax/CLAMS metabolic chamber system for ~24 hr, and data from a continuous 24-hr period was then recorded and analyzed.

### Gut microbial community DNA preparation

Approximately 20–50 mg of cecal matter was added to an autoclaved Sarstedt 2 m micro screw-cap tube (Ref# 72.693.005) containing approximately 500 μL of BioSpec Zirconia/Silica beads (Cat# 11079101z) and one large Bio Spec bead (Cat# 11079132ss). To this, 500 μL of 200 mM Tris-HCl, pH 8.0/200 mM NaCl/20 mM EDTA was added, as well as 210 μL 20% SDS. 500 μL of Phenol/Chloroform/isoamyl alcohol, pH 7.9, 25:24:1, was added before bead beating using a FastPrep 24 (M.P. Biomedicals) until sample was fully homogenized in solution. Tubes were centrifuged at 8,000 rpm at 4 °C for three minutes. The aqueous layer, approximately 500 µL, was transferred to a new microcentrifuge tube (Axygen). 60 μL of 3 M NaAcetate was added, then 600 μL of isopropanol, then inverted to mix. The samples were placed on ice for one hour before centrifuging at 13,000 rpm at 4 °C for 20 minutes. Samples were decanted, and pellet was rinsed with 200 μL of 100% EtOH, then decanted and briefly dried. The pellet was dissolved in 100–200 μL of TE buffer. 100 μL of DNA was cleaned using the Macherey-Nagel PCR Clean-up kit, using 2 NT3 washes and eluting with 50–100 μL of elution buffer.

### Construction and Sequencing of v3-v4 16S Metagenomic libraries

Purified genomic DNA was submitted to the University of Wisconsin-Madison Biotechnology Center. DNA concentration was verified fluorometrically using either the Qubit® dsDNA HS Assay Kit or Quant-iT™ PicoGreen® dsDNA Assay Kit (ThermoFisher Scientific, Waltham, MA, USA). Samples were prepared in a similar process to the one described in Illumina’s 16 S Metagenomic Sequencing Library Preparation Protocol, Part # 15044223 Rev. B (Illumina Inc., San Diego, California, USA) with the following modifications: The 16 S rRNA gene V3/V4 variable region was amplified with fusion primers (forward primer 341 f: 5′-ACACTCTTTCCCTACACGACGCTCTTCCGATCT(N)_3/6_CCTACGGGNGGCWGCAG-3′, reverse primer 805r: 5′-GTGACTGGAGTTCAGACGTGTGCTCTTCCGATCT(N)_3/6_GACTACHVGGGTATCTAATCC-3′). Region specific primers were previously described (^43^; underlined sequences above), and were modified to add 3–6 random nucleotides ((N)_3/6_) and Illumina adapter overhang nucleotide sequences 5′ of the gene‐specific sequences. Following initial amplification, reactions were cleaned using a 0.7x volume of AxyPrep Mag PCR clean-up beads (Axygen Biosciences, Union City, CA). In a subsequent PCR, Illumina dual indexes and Sequencing adapters were added using the following primers (Forward primer: 5′-AATGATACGGCGACCACCGAGATCTACAC[55555555]ACACTCTTTCCCTACACGACGCTCTTCCGATCT-3′, Reverse Primer: 5′-CAAGCAGAAGACGGCATACGAGAT[77777777]GTGACTGGAGTTCAGACGTGTGCTCTTCCGATCT-3′, where bracketed sequences are equivalent to the Illumina Dual Index adapters D501-D508 and D701-D712,N716,N718-N724,N726-N729). Following PCR, reactions were cleaned using a 0.7x volume of AxyPrep Mag PCR clean-up beads (Axygen Biosciences). Quality and quantity of the finished libraries were assessed using an Agilent DNA 1000 kit (Agilent Technologies, Santa Clara, CA) and Qubit® dsDNA HS Assay Kit (ThermoFisher Scientific), respectively. Libraries were pooled in an equimolar fashion and appropriately diluted prior to sequencing. Paired end, 300 bp sequencing was performed using the Illumina MiSeq Sequencer and a MiSeq. 600 bp (v3) sequencing cartridge. Images were analyzed using the standard Illumina Pipeline, version 1.8.2. OTU assignments and diversity plots were created using QIIME analysis pipeline^44^.

### Microbiota analysis using QIIME

Microbiota analysis to obtain OTU assignments and diversity plots were performed using Quantitative Insights Into Microbial Ecology (QIIME)^44^ version 1.9.1. Illumina sequencing reads were adapter and quality trimmed using the Skewer^45^ trimming program to remove low quality (<Q25) bases and sequencing adapters. Reads shorter than 100 nucleotides after trimming were discarded. Flash^46^ was used to merge paired end reads into amplicon sequences using a minimum overlap of 10 nucleotides. Amplicons were then PCR primer trimmed and quality filtered. Sequences were then clustered in OTUs using an open-reference OTU picking protocol based on 97% identity using UCLUST^46^ against the Greengenes reference database^47^. Representative sequences (most abundant sequence in OTUs) were picked, aligned to GreenGenes^47^ Core reference alignment using PyNAST^48^. Taxonomic assignments were associated with OTUs based on the taxonomy associated with the Greengenes reference sequence defining each OTU. UniFrac distances between samples were calculated using the Greengenes reference tree (ftp://greengenes.microbio.me/greengenes_release/gg_13_5/gg_13_8_otus.tar.gz). The resulting biom-formatted OTU table was filtered to remove singletons and OTUs that could not be aligned using PyNAST. Alpha rarefaction curves were calculated for all samples with a rarefaction upper limit of (median depth/sample count). Samples were removed from further characterization if they did not contain sufficient reads at a depth where the Good’s coverage value for most samples was greater than 0.9. Beta diversity was calculated using weighted and unweighted unifrac on OTU data leveled according to the lowest sample depth. An alternative normalization by CSS^49^ is also provided for additional downstream analysis.

### Liver mRNA preparation and transcriptional profiling

Total liver RNA was extracted with Trireagent (Sigma) as previously described^19^. Concentration and purity of RNA was initially determined using a Nanodrop 2000c, and then submitted to the University of Wisconsin- Madison Biotechnology Center Gene Expression Center & DNA Sequencing Facility. RNA quality was then assayed using an Agilent RNA NanoChip, and stranded mRNA libraries with polyA enrichment were prepared as described in the Illumina TruSeq Stranded mRNA Sample Preparation Guide Rev. E. DNA sequencing was performed using an Illumina HiSeq. 2500 1 × 100 (TruSeq v3) full flowcell. Pathway enrichment was performed using the “edgeR”^50^ and “org.Mm.eg.db”^51^ packages in R version 3.4.4^52^.

### Gene expression (qPCR)

Total RNA was isolated from liver with Tri-Reagent, and cDNA was generated as previously described^24^. Oligo dT primers and primers for real-time PCR were obtained from Integrated DNA Technologies (Coralville, IA,USA). Primer sequences used for qPCR were as follows: *Cyp8b1*: F: GTTTCTGGGTCCTCTTATTCCTG, R: TGGGAGTGAAAGTGAACGAC; *Zfp36l1*: F: CACACCAGATCCTAGTCCTTG, R: CTGGGAGTGCTGTAGTTGAG; *Cyp7a1*: F: AACGATACACTCTCCACCTTT, R: CTGCTTTCATTGCTTCAGGG; *Ppp2cb*: F: ATGGAAGGATATAACTGGTGCC, R: AGGTGCTGGGTCAAACTG; *Map2k1*: F: CGTACATCGTGGGCTTCTAC, R: CAGAACTTGATCCAAGGACCC; *Shp*: F: CTACCCTCAAGAACATTCCAGG, R: CACCAGACTCCATTCCACG; *Abcb11:* F: CCTCATACGGAAACCCAAGATC, R: CTGACTGTTGATAGGCGATGG; *Actb*: F: ACCTTCTACAATGAGCTGCG, R: CTGGATGGCTACGTACATGG. Reactions were run on an Applied Biosystems StepOnePlus Real-Time PCR System (Thermo Fisher Scientific) with Invitrogen SYBR Green PCR Master Mix (ThermoFisher Scientific). Actin (*Actb*) was used to normalize the results from gene-specific reactions.

### Statistics

Statistics were carried out in Prism 7 (Graphpad Prism). For each measured parameter, we conducted a two‐factor ANOVA which included an effect of drug treatment (vehicle or antibiotics), an effect of diet (Control or Low AA), and an interaction between diet and treatment. A Sidak’s post-test was performed to determine the significance of factors identified as significant in the two-factor ANOVA. PCA analysis was performed using Clustvis^53^.

## Results

### A Low Amino Acid Diet Alters the Taxonomic Composition of the Gut Microbiome

**Figure 1:**
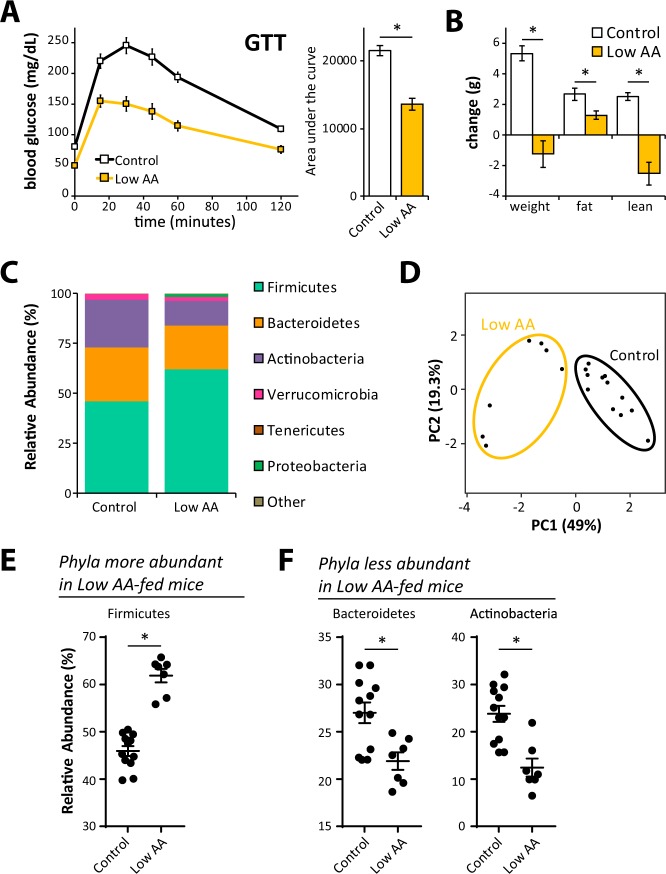
A low protein diet promotes metabolic health and alters the taxonomic composition of the cecal microbiome. (**A**) Glucose tolerance test on male C57BL/6J mice fed a Control (22% of calories from amino acids) or Low AA (7% of calories from amino acids) diet for 4 months (n = 8–10/group; *p < 0.05, t-test). (**B**) Weight and body composition were measured immediately prior to diet start and after 10 weeks on the indicated diets (n = 8–10/group; *p < 0.05, = t-test). (**C**) Bar plot of average relative abundance at the phylum taxonomic level. Top 6 phyla are shown. (**D**) Principle component analysis of demonstrating the effect of diet on taxonomic composition. (**E**,**F**) Bacterial phyla differentially represented in cecal contents from mice fed the specified diets for 4 months (n = 7–12/group; Sidak’s test following ANOVA, *p < 0.05). Error bars represent SEM.

To determine if the taxonomic composition of the microbiome is altered by consuming reduced dietary protein, we fed mice amino acid (AA) defined diets modeled on the AA profiles of naturally sourced control and low protein diets. The Control diet is modeled on a naturally sourced 21% protein diet, while the Low AA diet is based on a naturally sourced 7% protein diet, and we have previously shown that low protein and Low AA diets are comparable in their effect on glycemic control and body composition^22^. As shown in Fig. 1, mice fed a Low AA diet for four months have improved glucose tolerance (Fig. 1A) and reduced weight and fat mass gain relative to mice fed the Control diet (Fig. 1B).

We sacrificed mice fed the Control and Low AA diets after four months, collecting cecal contents and the liver. We prepared DNA from the contents of the cecum, and performed 16 S ribosomal RNA (rRNA) sequencing in order to identify alterations in the microbial composition of the gut microbiome. We utilized QIIME to determine relative taxonomic composition of each sample at the phylum and family levels (Figs 1C and S1A). While the alpha diversity did not significantly differ between Control and Low AA diet-fed mice (Fig. S1B), we observed a major shift in the taxonomic composition of the gut microbiome. Utilizing principal component analysis, we determined that the first two principle components explained a majority of the variability in the taxonomic composition of the gut microbiome, and individuals clustered by diet (Fig. 1D). We observed major differences at the phylum level, with an increase in the relative abundance of Firmicutes (Fig. 1E), and a decrease in the relative abundance of Bacteroidetes and Actinobacteria (Fig. 1F). At the family level, we found that the increased relative abundance of Firmicutes was primarily driven by an increase in the order *Clostridiales* (Fig. S1c); we also observed a decrease in the abundance of the Bacteroidetes families *S24–7* and *Odoribacteraceae*, and the Actinobacteria families *Bifidobacteriaceae* and *Coriobacteriaceae* (Fig. S1D).

### A Low Amino Acid Diet Alters the Hepatic Transcriptome, but does not activate FXR-FGF15 signaling

A low protein diet improves glucose tolerance in part by improving hepatic insulin sensitivity^22,54^. While induction of the insulin-sensitizing hormone FGF21 has been shown to play a role in this response^20,21^, possibly via inhibition of hepatic mTORC1 (mechanistic Target Of Rapamycin Complex 1)^55^, we recently observed that dietary methionine restriction, which mimics many of the effects of a low protein diet, can improve glucose tolerance independently of changes in FGF21 and hepatic mTORC1^24^.

Over the past decade, it has become clear that the composition and function of the gut microbiome can regulate host metabolism, including glycemic control^56,57^. One recently characterized pathway by which the microbiome can regulate host glucose metabolism is through bile acids; alterations in the amount and type of secondary bile acids can regulate glycemic control by activating or repressing the FXR – FGF15 signaling axis^34,35^.

**Figure 2:**
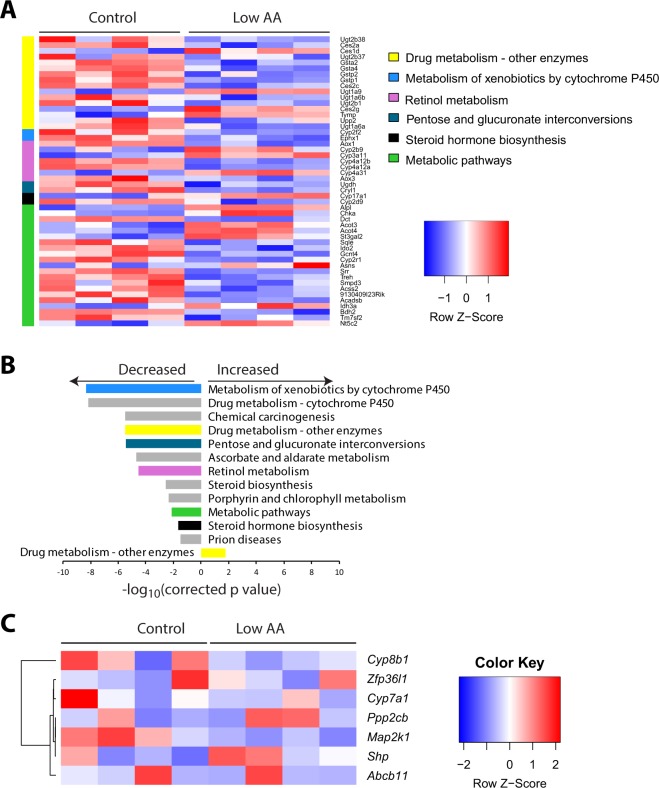
A low protein diet alters the hepatic transcriptome and shows distinct changes in biological pathways. (**A**–**C**) RNA-Seq was performed on the livers for mice fed a Control diet or a Low AA for four months. (**A**) A heatmap indicating the relative expression of genes involved in the most significantly enriched biological KEGG (Kyoto Encyclopedia of Genes and Genomes) pathways based on genes differentially expressed in the livers of Control and Low AA fed mice (q < 0.05, FDR). Genes in more than one significantly enriched KEGG pathway are listed only once, and assigned to the most significantly affected pathway. (**B**) Pathway enrichment analysis was performed using g:Profiler (g:GOSt)^73^, and the p-values of KEGG pathways significantly up- and downregulated by Low AA diet feeding were determined. Colors are matched to that of pathways in (**A**). (**C**) Heatmap representing the relative expression of liver genes known to be altered by FXR-FGF15 bile acid signaling.

We used RNA-Seq to identify gene expression changes induced by a low protein diet. We identified several differentially expressed metabolic pathways when mice were fed a Low AA diet (Fig. 2A,B). In particular, we observed altered expression of many genes involved in xenobiotic or drug metabolism as well as steroid hormone biosynthesis. However, our analysis did not identify bile acid signaling as significantly altered, and we did not observe significantly altered expression of *Shp*, *Cyp7a1*, or several other genes that have been shown to be regulated by the FXR-FGF15 signaling axis (Fig. 2C)^58–60^. Targeted qPCR analysis of mRNA from a larger, additional cohort of mice confirmed that a Low AA diet did not significantly alter the expression of any of one of these genes (Fig. S2). However, we observed an overall effect of diet consistent with reduced FXR-FGF15 signaling (Fig. S2).

### The metabolic effects of protein restriction are not mediated by the gut microbiota

**Figure 3:**
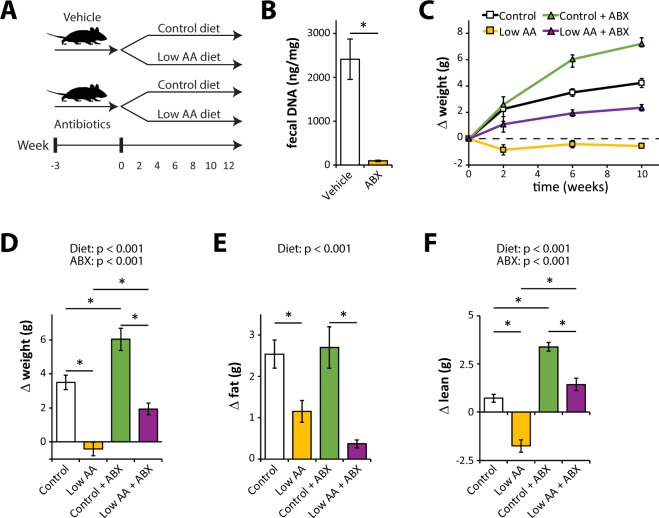
A low protein diet alters body composition similarly in vehicle and antibiotic-treated mice. (**A**) Schematic representation of the experimental plan; mice were pretreated with antibiotics or vehicle for three weeks, and then randomized to either a Control or Low AA diet. (**B**) Fecal DNA content was determined following 3 weeks of antibiotic treatment (n = 8/group; *p < 0.05, t-test). (**C**) Weight of the mice in each group was tracked following randomization to each diet. (**D**–**F**) Weight and body composition were determined immediately prior to diet start and after 6 weeks on the indicated diets, and the change in (**D**) weight, (**E**) fat mass, and (**F**) lean mass was determined (n = 12/group; statistics for the overall effects of diet, antibiotic (ABX) treatment, and the interaction represent the p-value from a two-way ANOVA; *p < 0.05 from a Sidak’s post-test examining the effect of parameters identified as significant in the two-way ANOVA). Error bars represent SEM.

In order to directly assess if the altered taxonomic composition of the gut microbiome contributes to the metabolic effects of a low protein diets, we pretreated mice with either vehicle or antibiotics (ABX) for three weeks; mice were then randomized to either Control or Low AA diets. Antibiotic-treated mice continued to receive antibiotics throughout the course of the experiment (Fig. 3A). As expected, mice treated with antibiotics had significantly reduced fecal DNA content (Fig. 3B). Over the course of the experiment, we tracked weight and body composition of mice in each group (Fig. 3C–F). As expected based on our previous studies, mice fed the Low AA diet gained less weight, less fat mass, and less lean mass than mice fed a Control diet. While antibiotic administration increased weight gain and lean mass gain compared to vehicle treated mice, the Low AA diet had similar effects on weight and body composition in the presence and absence of antibiotics.

**Figure 4:**
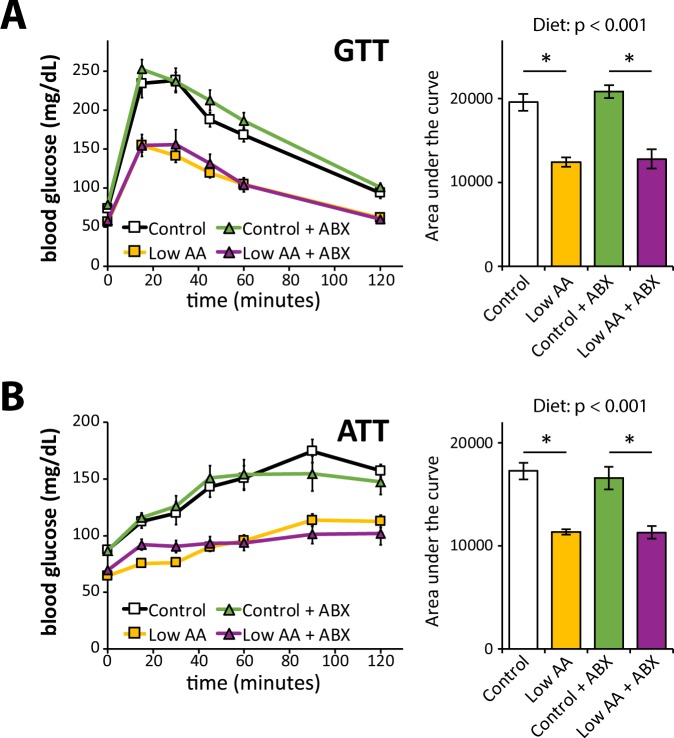
A low protein diet improves glucose homeostasis similarly in vehicle and antibiotic-treated mice. (**A**) Glucose and (**B**) alanine tolerance tests were conducted in mice fed the indicated diets for 8 weeks and 4 weeks, respectively (n = 12/group; statistics for the overall effects of diet, antibiotic (ABX) treatment, and the interaction represent the p-value from a two-way ANOVA; *p < 0.05 from a Sidak’s post-test examining the effect of parameters identified as significant in the two-way ANOVA). Error bars represent SEM.

As we determined previously, mice fed a Low AA diet had improved glucose tolerance compared to mice fed a Control diet (Fig. 4A). We also specifically assessed gluconeogenesis in the liver by performing an alanine tolerance test; as in our previous studies utilizing pyruvate, we observed a decrease in the area under the curve (AUC) in mice fed a Low AA diet, indicating improved suppression of gluconeogenesis (Fig. 4B). We saw equivalent reductions in AUC in response to a Low AA diet in both vehicle and antibiotic fed mice; and we did not observe any differences in AUC resulting from antibiotic treatment.

**Figure 5:**
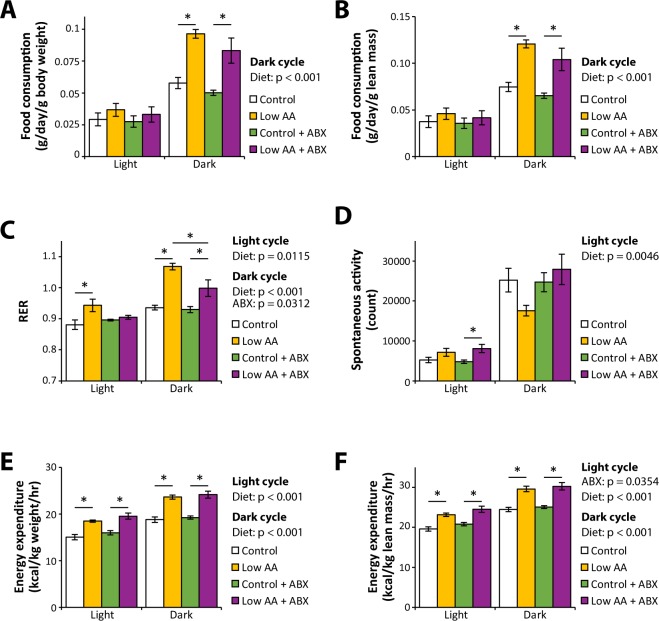
A low protein diet increases food consumption and energy expenditure similarly in vehicle and antibiotic-treated mice. (**A**–**F**) Metabolic chambers were used to assess (**A**,**B**) food consumption, (**C**) spontaneous activity, (**D**) respiratory exchange ratio (RER), and (**E**,**F**) energy expenditure in mice fed the indicated diets for approximately two months. (n = 5–7/group; statistics for the overall effects of diet, antibiotic (ABX) treatment, and the interaction represent the p-value from a two-way ANOVA; *p < 0.05 from a Sidak’s post-test examining the effect of parameters identified as significant in the two-way ANOVA). Error bars represent SEM.

Rodents fed a low protein diet have increased food consumption and increased energy expenditure^15,18,20–22,61^. We examined the effect of antibiotics on these phenotypes by placing mice in metabolic chambers and assessing food consumption, spontaneous activity, respiratory exchange ratio (RER), and energy expenditure (Fig. 5A–F). In agreement with our previous results, we observed increased food consumption (Fig. 5A,B) and energy expenditure (Fig. 5E,F) in mice fed a Low AA diet, with equivalent effects in both vehicle and antibiotic treated mice. Surprisingly, while a Low AA diet increased both the daytime and nighttime RER of vehicle-treated mice, mice treated with antibiotics did not have increased daytime RER when fed a Low AA diet, and had a reduced increase in nighttime RER (Fig. 5C). The effects of diet on spontaneous activity were relatively small, with increased activity in Low AA fed mice during the day (Fig. 5D).

## Discussion

Understanding the mechanisms by which dietary choices impact metabolic health is an area of significant research interest due to the increasing prevalence of diabetes and obesity in the population. Recently, we and others have shown that reducing dietary protein can promote metabolic health in both humans and rodents, but the molecular mechanisms that mediate these effects are unclear.

Here, we examine the effect of a low protein diet on the gut microbiome. In general agreement with the results of Holmes and colleagues^36^, we find that reducing dietary amino acid levels (and increasing dietary carbohydrates) results in an increased Firmicutes-to-Bacteroidetes ratio. These findings demonstrate the robustness of this intervention on the gut microbiome, as the same effect can be observed in two different laboratories on different continents, using either naturally sourced or defined diets. These results are somewhat surprising, as some previous studies have suggested a link between obesity and an increased prevalence of Firmicutes^62^. However, they are consistent with the model of Holmes and colleagues that increased caloric intake promotes Firmicutes abundance^36^.

Since the gut microbiome has proven to be a potent regulator of metabolic health, obesity, and glycemic control, we tested the hypothesis that diet-induced changes in the gut microbiome mediate the beneficial effects of a low protein diet on metabolism. We first used a targeted approach, examining the response of the liver, which is responsive to microbiome-mediated alterations in the amount and type of secondary bile acids via the FXR-FGF15 signaling axis^34,35^, at the transcriptional level. While we observed altered expression of many genes in response to reduced dietary protein, we found no evidence of altered bile acid signaling and no evidence of increased signaling by the FXR-FGF15 signaling axis. However, a follow-up qPCR analysis of a targeted panel of genes regulated by FXR-FGF15 signaling in a second cohort of mice suggested that a low protein diet might reduce hepatic FXR-FGF15 signaling.

To directly test the role of gut microbiome, we then took an unbiased approach, testing the requirement for an intact gut microbiome in the metabolic response to reduced dietary protein. Following three weeks of high-dose antibiotic treatment, a regimen previously shown to ablate the gut microbiome and dramatically reduce fecal DNA content, we placed mice on either Control or Low AA diets. As anticipated based on previous studies in mice and many other mammals^63–66^, antibiotics had an overall positive effect on growth and lean mass. However, we observed that protein restriction had very similar effects on weight, body composition, glucose tolerance, and energy expenditure in both the presence and absence of antibiotics. The one major difference we observed was that antibiotic treated, Low AA-fed mice had a lower RER relative to their vehicle treated counterparts, suggesting increased utilization of lipids and decreased utilization of carbohydrates as a fuel source.

A significant caveat of our studies is that they were conducted in young, lean C57BL/6 J mice, which have relatively low intestinal permeability; this may limit the ability of the gut microbiome composition to affect host metabolism. The microbiome could play a larger role in the metabolic response of obese or older animals, which have increased gut permeability^67,68^; therefore, investigating the role of the gut microbiome in response to reduced dietary protein in aged or obese animals might be an important area for future study. In addition, we did not examine the taxonomic composition of the gut microbiome of antibiotic treated mice, which could help clarify if there are any antibiotic-resistant microbes which might mediate the metabolic effects of a low protein diet. Finally, we did not examine the metabolic effects of protein restriction in germ-free mice; examination in these animals, which completely lack all bacteria, could conceivably reveal subtle effects of the gut microbiome on the response to dietary protein that were not detectable using our antibiotic-ablation model.

Our study also examined only a limited number of metabolic phenotypes associated with a low protein diet, over a relatively short period of time; other phenotypes associated with reduced protein consumption, including increased longevity and healthspan^69^, may be more directly linked to composition of the gut microbiota. A more detailed investigation of the transcriptional response to protein restriction in antibiotic-treated or germ-free mice may provide valuable clues to identify specific phenotypes that are dependent upon changes in the gut microbiome. There is also growing understanding that the specific amino acid composition of the diet mediates metabolic health^70–72^, and there may be a role for the microbiome in the metabolic response to other diets with unusual amino acid profiles or from particular dietary sources.

Our findings highlight that dietary macronutrient composition plays an important role in determining the taxonomic composition of the gut microbiome. Yet, while the effects of a low protein diet on the gut microbiome are dramatic, at least in the short term, an intact gut microbiome is not required to realize the metabolic benefits of a low protein diet on glucose homeostasis, body composition, or energy balance. Identifying the physiological and molecular mechanisms by which reducing dietary protein can promote metabolic health remains critical to developing drugs which can take advantage of these pathways to combat obesity and diabetes.

## Supplementary information

Supplementary Information

## Data Availability

Liver transcriptional profiling data has been deposited at GEO, accession number GSE115683. All other datasets generated during the current study are available from the corresponding author on reasonable request.
