# Supplementary material for: The Metabolic Response to a Low Amino Acid Diet is Independent of Diet-Induced Shifts in the Composition of the Gut Microbiome
Source: Sci Rep. 2019 Jan 11;9:67. doi: 10.1038/s41598-018-37177-3 (PMC6329753; doi:10.1038/s41598-018-37177-3)
Supplement: Supplementary file 1 — Supplementary Information [file 41598_2018_37177_MOESM1_ESM.pdf]

The Metabolic Response to a Low Amino Acid Diet is Independent of Diet-Induced Shifts in the Composition of the Gut Microbiome

Heidi H. Pak, Nicole E. Cummings, Cara L. Green, Jacqueline A. Brinkman, Deyang Yu, Jay L. Tomasiewicz, Shany E. Yang, Colin Boyle, Elizabeth N. Konon, Irene M. Ong, Dudley W. Lamming

Supplemental Legends

Figure S1: A) Bar plot of average relative abundance at the family taxonomic level. Top 10 phyla are shown. **B)** Bacterial alpha rarefaction curves calculated according to Faith's phylogenetic diversity (PD) index at the phylum taxonomic level. **C-D)** Bacterial families differentially represented in cecal contents from mice fed the specified diets for 4 months (n= 7-12/group; $q < 0.05$, FDR and * = $p < 0.05$, t-test). Error bars represent SEM.

Figure S2: qPCR analysis of FXR-FGF15 controlled genes. mRNA expression of the indicated genes in the livers of mice fed Control or Low AA diets was assessed by qPCR (n=7-8/group; statistics for the overall effects of gene, diet and the interaction represent the p-value from a two-way ANOVA; * = $p < 0.05$ from a Sidak’s post-test examining the effect of parameters identified as significant in the two-way ANOVA). Error bars represent SEM.

Table S1: List of differentially expressed genes contributing to the identification of KEGG Pathways in Figure 2.

Figure S1

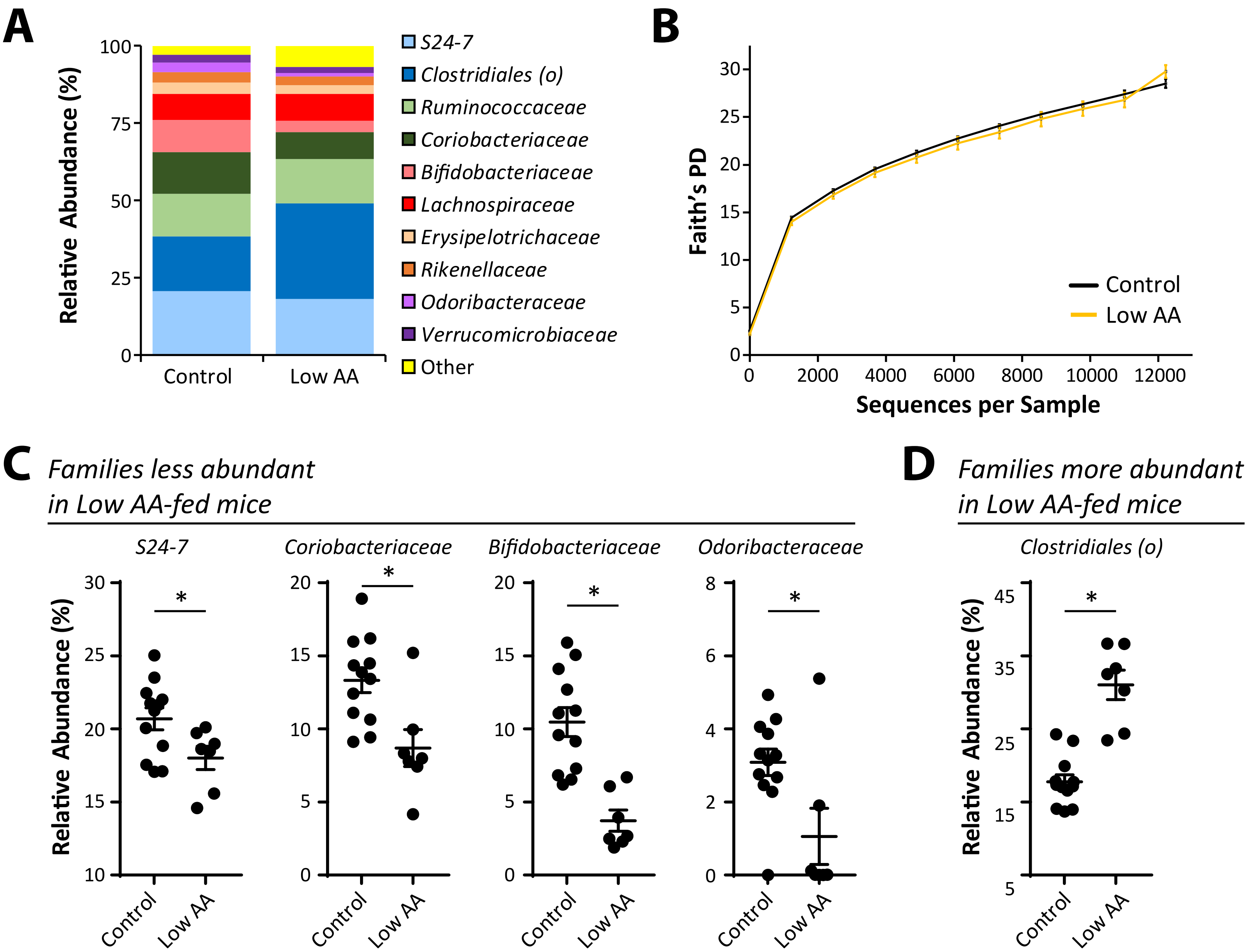

Figure S2

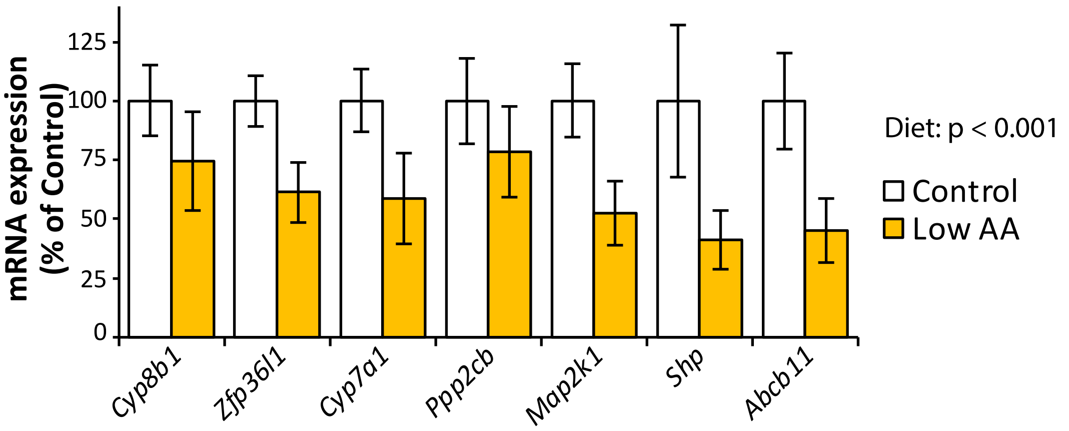

Table S1

KEGG Pathway	Gene list
Metabolism of xenobiotics by cytochrome P450	GSTA4,UGT2B1,ENSMUSG00000038155,EPHX1,CYP2F2,UGT1A6A,UGT2B37,GSTA2,ENSMUSG00000060803,UGT2B38,UGT1A6B
Drug metabolism - cytochrome P450	GSTA4,UGT2B1,ENSMUSG00000038155,UGT1A6A,UGT2B37,GSTA2,ENSMUSG00000060803,UGT2B38,AOX1,AOX3,UGT1A6B
Chemical carcinogenesis	GSTA4,UGT2B1,ENSMUSG00000038155,EPHX1,UGT1A6A,UGT2B37,GSTA2,ENSMUSG00000060803,UGT2B38,UGT1A6B
Drug metabolism - other enzymes	UPP2,UGT2B1,UGT1A6A,CES2A,UGT2B37,CES2C,UGT2B38,UGT1A6B
Pentose and glucuronate interconversions	CRYL1,UGDH,UGT2B1,UGT1A6A,UGT2B37,UGT2B38,UGT1A6B
Ascorbate and aldarate metabolism	UGDH,UGT2B1,UGT1A6A,UGT2B37,UGT2B38,UGT1A6B
Retinol metabolism	UGT2B1,UGT1A6A,UGT2B37,UGT2B38,AOX1,AOX3,CYP4A12A,CYP4A12B,UGT1A6B
Steroid biosynthesis	SQLE,TM7SF2,CYP2R1,MSMO1
Porphyrin and chlorophyll metabolism	UGT2B1,UGT1A6A,UGT2B37,UGT2B38,UGT1A6B
Metabolic pathways	SRR,ETNPPL,CRYL1,DCT,SQLE,TM7SF2,UPP2,ACSS2,BDH2,UGDH,CYP2R1,ACADSB,IDO2,MSMO1,SMPD3,TREH,TKFC,UGT2B1,9130409I23RIK,UGT1A6A,UGT2B37,UGT2B38,AOX1,AOX3,CYP4A12A,CYP4A12B,UGT1A6B,GCNT4
Steroid hormone biosynthesis	UGT2B1,UGT1A6A,UGT2B37,UGT2B38,CYP2D9,UGT1A6B
Prion diseases	C9,C6,NCAM2,PRNP
Drug metabolism - other enzymes	TYMP,CES2G,CES1D,UGT1A9
